# Antioxidant Activities of *Solanum nigrum* L. Leaf Extracts Determined in In Vitro Cellular Models

**DOI:** 10.3390/foods8020063

**Published:** 2019-02-08

**Authors:** Agata Campisi, Rosaria Acquaviva, Giuseppina Raciti, Anna Duro, Milena Rizzo, Natale Alfredo Santagati

**Affiliations:** 1 Department of Drug Science, University of Catania, Viale Andrea Doria 6, 95125 Catania, Italy; agcampisi@gmail.com (A.C.); racquavi@unict.it (R.A.); racitigi@unict.it (G.R.); santagat@unict.it (N.A.S.); 2 Department of Biological, Geological and Environmental Sciences, University of Catania, Via A. Longo 19, 95125 Catania, Italy; annaduro@unict.it

**Keywords:** *Solanum nigrum* L. leave extracts, natural products, antioxidant activity, functional food

## Abstract

Several medicinal foods abound in traditional medicine with antioxidant potentials that could be of importance for the management of several diseases but with little or no scientific justification to substantiate their use. Thus, the objective of this study was the assessment of the antioxidant effect of two leave extracts of *Solanum nigrum* L. (SN), which is a medicinal plant member of the *Solanaceae* family, mainly used for soup preparation in different parts of the world. Then methanolic/water (80:20) (SN1) and water (SN2) leaves extracts were prepared. The total polyphenolic content and the concentration of phenolic acids and flavones compounds were determined. In order to verify whether examined extracts were able to restore the oxidative status, modified by glutamate in primary cultures of astrocytes, the study evaluated the glutathione levels, the intracellular oxidative stress, and the cytotoxicity of SN1 and SN2 extracts. Both extracts were able to quench the radical in an in vitro free cellular system and restore the oxidative status in in vitro primary cultures of rat astroglial cells exposed to glutamate. These extracts prevented the increase in glutamate uptake and inhibited glutamate excitotoxicity, which leads to cell damage and shows a notable antioxidant property.

## 1. Introduction

Several medicinal foods abound in traditional medicine with antioxidant activities. This could be of important for the management of several diseases but has little or no scientific justification to substantiate their use. In the tropics, as in Asia and in sub-Saharan Africa, green leafy vegetables are used as one of the major components of local dishes. 

*Solanum nigrum* L. (SN) belongs to the *Solanaceae* family to Europe, Asia, and North America and was introduced in South America, Australia, and Africa. It represents one of the largest and most variable species groups of the genus. SN, well known as “Black Nightshade” (the English name), is an herbal plant widely distributed throughout the world, extending from tropical regions to temperate regions [1].

In many developing countries, SN constitutes a minor food crop, with the shoots and berries not only used as vegetables and fruits but also for various medicinal and local uses [2]. SN serves mainly as vegetables for soup preparation in different parts of the world. Several studies have investigated the nutritive value of the ‘vegetable black nightshade,’ which put forward evidence that this species constitutes a nutritious vegetable [3]. This plant was chosen not only for being nutritive, but also for their folkloric reports of medicinal properties [4]. Studies document potential health benefits of different parts of this vegetable. SN leaves have been reportedly used in traditional medicine for the management of several diseases including seizure and epilepsy, pain, ulcer, inflammation, diarrhea, some eye infections, and jaundice [5,6].

In folklore medicine, the leaves are used to treat oral ulcers in India where an interesting pharmacological investigation has been performed by using an aqueous extract of SN leaves [7].

More recently, many research studies have reported that SN showed anti-cancer activity for hepatocellular carcinoma cells [8], human ovarian carcinoma cells [9], human colorectal carcinoma cells [10], and human endometrial carcinoma cells [11]. The leaves can provide appreciable amounts of protein and amino acids, minerals including calcium, iron, and phosphorus, vitamins A and C, fats and fibers, and appreciable amounts of methionine, which is an amino acid scarce in other vegetables. Other chemical constituents reported in leaves are steroidal glycosides [12]. Very recently, from the unripe berries, a previously undescribed steroidal alkaloids [13] and steroidal glycosides [14] were isolated. Those compounds showed a potent inhibitory activity against the lipopolysaccharide (LPS)-induced nitric oxide (NO) production.

Because medicinal plants are gaining popularity for the production of reliable and safe medicines suitable for human, many studies investigated the composition of extracts, their biological activities, and optimization of extraction procedures [15,16].

The extracts of the SN contain many polyphenolic compounds. The leaves are rich in polyphenols, including phenolic acids and flavones [17]. Zaidi et al. demonstrated that treatment of rats with SN leaves extract was able to reduce oxidative stress, and, in particular, they showed the potential of this extract in preventing/alleviating stress-induced diseases, involving oxidative damage to cellular constituents especially the brain [18]. Antioxidant activity might be due to the presence of the above-mentioned polyphenolic compounds on SN stems and leaves [19]. Sun et al. demonstrated that oxidative stress has been associated with pathological conditions, including Central Nervous System (CNS) diseases and physiological brain aging processes [20].

A very interesting study has shown that dietary inclusions of Solanum leaf could protect against cognitive and neurochemical impairments induced by scopolamine, and, hence, this vegetable could be used as a source of functional foods and nutraceuticals for the prevention and management of cognitive impairment-associated diseases such as Alzheimer’s disease [21].

The formation and release of Radical Oxygen reactive Species (ROS) cause structural and functional alterations of cell membranes. Free radicals attack polyunsaturated fatty acids in bio-membranes and mitochondria begin the main source of ROS, when the mitochondrial respiratory chain is impaired. In these cases, a compound possessing antioxidant properties can be useful in stopping ROS production and limiting oxidative cell damages, which is particularly interesting if this activity is produced by a functional food [22]. Experimentally, ROS is well determined by using reduced glutathione (GSH), which is known as the most important scavengers of reactive species, and a reduced glutathione/oxidized glutathione ratio is used as a marker of oxidative stress [23].

At the central level, the oxidative stress may activate several calcium-dependent enzymes, causing mitochondria impairment, a decrease in adenosine triphosphate (ATP) levels, ROS production, and subsequent neuronal cell death [24]. A brief exposure to glutamate, which is a major excitatory neurotransmitter in the CNS, could determine several acute and chronic brain damages on differentiated astrocytes, which then causes cell swelling, whereas a prolonged incubation (excitotoxicity) induces cell damage [25,26]. This phenomenon causes alterations in glutamate transport, GSH depletion, and macromolecular synthesis [27].

Herein, we prepared two SN polar leaf extracts and assessed the total polyphenolic content and the concentration of phenolic acids and flavones compounds. Antioxidant activity in both an in vitro cellular free system and in vitro cellular system was evaluated. To verify whether SN1 and SN2 extracts were able to restore the oxidative status, which were modified by glutamate in primary cultures of astrocytes, GSH, ROS levels and the cytotoxicity of both extracts has been assessed.

## 2. Materials and Methods

### 2.1. Materials

Reference compounds (gallic acid, protocatechuic acid, chlorogenic acid, gentisic acid, caffeic acid, luteolin, apigenin) were purchased from Sigma (St. Louis, MO, USA). Acetonitrile, methanol, and water were chromatographic-grade and were bought from Carlo Erba (Milano, Italy). STable 2,2-diphenyl-1-picrylhydrazyl (DPPH) radical, 3(4,5-dimethyl-thiazol-2-yl)2,5-diphenyl-tetrazolium bromide (MTT), 2′,7′-dichlorofluorescein diacetate (DCFH-DA), 6-hydroxy-2,5,7,8-tetramethylchroman-2-carboxylic acid (Trolox), dimethylsulfoxide (DMSO), GYKI 52466, phosphate buffer solution (PBS), and other analytical chemicals were purchased from Sigma-Aldrich Chimica (Milan, Italy). Distilled and deionized water was used for the preparation of all samples and solutions and they were used after filtration through HA filters (0.45 µm Millipore, Bedford, MA, USA). All standards were diluted to the appropriate obtained concentration and were stored at +4 °C in amber vials in a dark place until analysis. Dulbecco′s modified Eagle′s medium (DMEM) and heat-inactivated Fetal Bovine Serum (FBS) were obtained from Invitrogen (Milan, Italy). The mouse monoclonal antibody against glial fibrillary acidic protein (GFAP) and anti-IgG polyclonal antibody were from Chemicon (Prodotti Gianni, Milan, Italy).

### 2.2. Methods

#### 2.2.1. Plant Material and Extraction Procedures

SN leaves were collected around Catania (Sicily, Italy) in June 2018 and air-dried at room temperature (24 ± 2 °C) with no direct light. A voucher specimen of the plant has been deposited (N. 8234) in the herbarium of the Botany Department of the University of Catania (Italy). The dried and powdered leaves (5 g) were extracted by maceration for 24 h at room temperature with 150 mL each of methanol/water solution 80:20 *v*/*v* (SN1) and water (SN2), respectively. The extraction procedure was repeated three times, and the solvent extracts were combined and separated from the residue by filtration through Whatman N. 1 filter paper in a Buchner funnel under vacuum. The methanol was removed under a reduced pressure below 40 °C by using a rotary evaporator, and the aqueous phase remaining after evaporation of the organic phase was freeze-dried. The water extract was freeze-dried. The obtained dried extracts were 1.305 ± 0.24 g for SN1 and 0.975 ± 0.31 g for SN2, respectively. The obtained dried extracts were stored at −20 °C until undergoing assays.

#### 2.2.2. Determination of Total Phenolic Content

The amount of total phenolic in the studied extracts was determined using a modified Folin-Ciocalteu method [28]. Extracts were prepared at a concentration of 0.5 mg/mL. An aliquot of a known dilution of the extract was mixed with 5 mL Folin–Ciocalteu reagent (previously diluted 10-fold with deionized water) and was allowed to react for 6 min. Then, 4 mL (70 g/L) of sodium carbonate solution was added to test tubes. The tubes were vortexed for 20 s and allowed to stand for 90 min for color development. Absorbance was measured at 760 nm by using the Perkin Elmer UV–Vis Lambda 25 spectrophotometer (Perkin Elmer Italia spa, Monza, Italy). Extract samples were evaluated at the final concentration of 1 mg/mL. The measurements were compared to a standard curve of prepared gallic acid solutions. The total phenolic content were 92.2 mg/g of extract for SN1 and 40.0 mg/g of extract for SN2, which was expressed as a gallic acid equivalent.

#### 2.2.3. Chromatography 

Chromatographic analysis was performed by using high-performance liquid chromatography (HPLC) (Perkin Elmer, Norwalk, CT, USA) Series 200 pump equipped with an LC-235C Diode Array Detector (DAD), auto-sampler, and column oven. Chromatographic data were processed by using a Turbochrom Workstation software, version 6.1.2 (Perkin Elmer, Norwalk, CT, USA). Separation and determinations were accomplished on a 5 µm Hypersil ODS RP-18 column (250 × 4.6 mm, Supelco, Bellefonte, PA, USA) fitted with a guard column (Hypersil ODS RP-18, 5 µm particles, 10 × 4.6 mm, Supelco, Bellefonte, PA, USA). All the samples were filtered, through 0.45 µm membrane filter, and degassed by an ultrasonic bath before the injection. The procedure was performed, as previously described [17].

#### 2.2.4. Determination of Antioxidant Activity in an In Vitro Cellular Free System

The stable DPPH radical was used for the determination of free radical-scavenging activity of the extracts [29]. Because of its odd electron, the DPPH radical gives a strong absorption band at 517 nm in visible spectroscopy (deep violet color). As this electron becomes paired off in the presence of a free radical scavenger, the absorption vanishes, and the resulting decolorizing is stoichiometric with respect to the number of electrons taken up. The reaction mixture contained 86 μM DPPH radical and different concentrations of each extracts (0.025-0.5-0.1-0.2-0.4 mg/mL) in 1 mL of ethanol. After 10 min at room temperature, the absorbance at 517 nm was recorded [30]. Trolox (30 µM), water-soluble derivative of vitamin E, was used as a standard. The assay was performed in triplicates. 

#### 2.2.5. Determination of Antioxidant Activity within an In Vitro Cellular System

Primary cultures of astrocytes were prepared from new-born albino rat brains (from 1-day-old to 2-day-old Wistar strain rats) as described [31]. Cerebral tissues, after dissection and careful removal of the meninges, were mechanically dissociated through 82 μm pore sterile mesh (Nitex, Darmastadt, Germany). Isolated cells were suspended in DMEM, supplemented with 20% (*v*/*v*) FBS, 2 mM glutamine, streptomycin (50 mg/mL), and penicillin (50 U/mL), and plated at a density of 3 × 10^6^ cells/100 mm dishes and of 0.5 × 10^5^ cells/chamber of multi-chambered slides. Cells were maintained at 37 °C in a 5% CO_2_ and 95% air humidified atmosphere for two weeks. The medium was exchanged every three days. The low initial plating density of dissociated cells was meant to favor the growth of astrocytes with only a very little oligodendroglial and microglial cells contamination. Astroglial cell cultures were characterized at 14 days in vitro (DIV), when it is confluent, by immunofluorescence staining with GFAP. All experiments conformed to the guidelines of the local Ethical Committee (University of Catania, Italy), and were carried out in accordance with the EC Directive 86/609/EEC for animal experiments.

Astrocytes at 14 DIV were treated with glutamate (500 µM) for 24 h, as previously described [32]. Those cultures were treated with different concentrations of SN1 and SN2 (0.5 and 1 mg/mL), which were added 20 min before glutamate exposure. Four replicates were carried out for each sample. In a subset of experiments, to assess the inhibition of glutamate effects, the astroglial cell cultures were incubated 20 min prior to glutamate exposure, with GYKI 52466 (100 µM), the specific AMPA/KA receptor antagonist. 

Astroglial cell survival analysis was performed by the MTT reduction assay, which evaluated mitochondrial dehydrogenase activity, as previous reported [33,34]. Astrocytes were set up 0.5 × 10^5^ cells per well of a 96-multiwell, flat-bottomed, 200-μL micro plate. They were maintained at 37 °C in a humidified 5% CO_2_ and 95% air mixture [33]. At the end of treatment time, 20 μL of 0.5% MTT in (pH 7.4) PBS were added to each micro-well. After 1 h of incubation with the reagent, the supernatant was removed and replaced with of DMSO (200 μL). The optical density of each well was measured with a micro-plate spectrophotometer reader (Titertek Multiskan, Flow Laboratories, Helsinki, Finland) at l = 570 nm. 

#### 2.2.6. Glutathione Measurement

Astroglial cell cultures were scraped off and lysed in 50 µM sodium phosphate buffer (pH 7.4). The Bradford assay determined the protein concentration in cell extracts [32]. Then, a Hitachi U-2000 spectrophotometer (Hitachi, Tokyo, Japan) chemically determined the total glutathione intracellular content (GSH + GSSG), as described by Chen YH et al. [35].

#### 2.2.7. ROS Levels Determination

Reactive species determination was performed by using DCFH-DA as a fluorescent probe. Furthermore, 100 μM of DCHF-DA was dissolved in 100% methanol, added to the cellular medium, and the cells were incubated at 37 °C for 30 min. Under these conditions, the acetate group was not hydrolyzed [12]. After incubation, astroglial cell cultures were lysed and centrifuged at 10,000× *g* for 10 min. The fluorescence corresponding to the radical oxidized species 2′,7′-dichlorofluorescein (DCF) was monitored by measuring the excitation (λ = 488 nm) and emission (λ = 525 nm), using an F-2000 spectrofluorometer (Hitachi).

Values are expressed as a percentage of fluorescence intensity per mg protein *versus* control (% I.F/mg prot vs. control). Protein concentration was measured, according to the Bradford assay applied by Li Volti et al [32].

### 2.3. Statistical Analysis

All values are presented as means ± S.D. of five separate measurements. Statistical analysis was performed using one-way ANOVA, which was followed by the Newman-Keuls post-hoc test. Differences were considered statistically significant at * *p* < 0.05 and ** *p* < 0.001.

## 3. Results 

### 3.1. Analysis of SN Extracts

The total phenolic contents was assayed by a modified Folin-Ciocalteu method using gallic acid, and is reported in Table 1, with the physical characteristics and the dry weight yields of SN1 and SN2.

**Figure 1:**
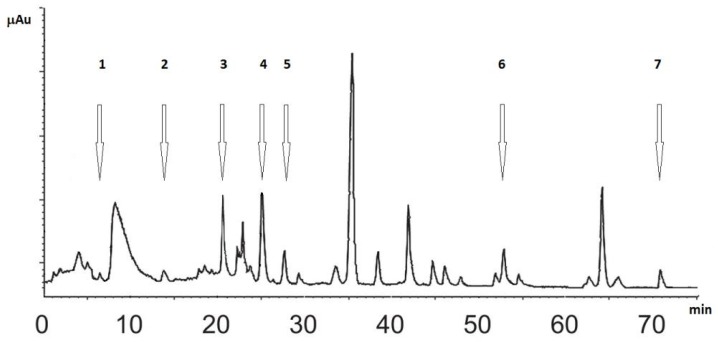
Representative chromatogram of the dry extract reporting the retention time (RT) of gallic acid (1, 0.65 min), protocatechuic acid (2, 13.85 min), chlorogenic acid (3, 20.5 min), gentisic acid (4, 25.1 min), caffeic acid (5, 27.5 min), luteolin (6, 52.9 min), and apigenin (7, 70.95 min). Axis: x label minutes, y label absorbance unit.

The relative concentrations of phenolic acids and flavones in SN1 and SN2 were determined by HPLC analysis, according to Huang et al. [29]. The values were expressed in mg/g of dry extract. The results of the five phenolic acids determination (gallic, protocatechuic, chlorogenic, gentisic, and caffeic) and two flavones (luteolin and apigenin) in studied extracts are summarized in Table 2 while Figure 1 shows a representative chromatogram.

It was found that the major compound in both extracts was chlorogenic acid, whereas gentisic acid is the second. Luteolin is more abundant than apigenin, and less amounts of caffeic acid and protocatechuic acid were found. Gallic acid exists only in traces together with other unknown compounds. 

### 3.2. Antioxidant Activity in a Cellular Free System

**Figure 2:**
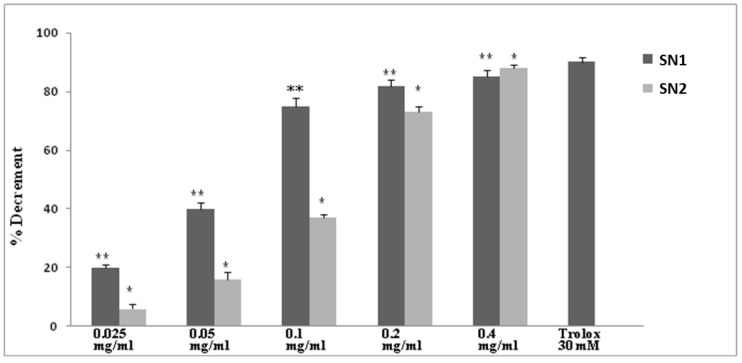
Quenching of DPPH of SN1 and SN2 extracts at different concentrations (0.025-0.5-0.1-0.2-0.4 mg/mL), compared to Trolox (30 mM). Axis: x label: concentration, y label: quenching of DPPH expressed as a percentage. (* *p* < 0.05 and ** *p* < 0.001).

The free radical-scavenging activity of SN1 and SN2 extracts was tested by their ability to bleach the stable DPPH radical. Both extracts were able to quench the DPPH-radical at all the concentrations used (0.025-0.5-0.1-0.2-0.4 mg/mL) in a dose-dependent manner. SN1 showed a more potent capacity than SN2. In addition, their effect appeared similar to Trolox (30 µM), which is a soluble analogous of vitamin E used as a standard. This experiment demonstrated that SN1 and SN2 possess antioxidant properties (Figure 2).

### 3.3. Antioxidant Activity in the Cellular System

Primary rat astroglial cultures exposed to 500 μM glutamate for 24 h were used as an in vitro cellular model to assess the antioxidant effect of SN1 and SN2 extracts. Cells were characterized by immuno-fluorescent staining using GFAP as a marker [31].

**Figure 3:**
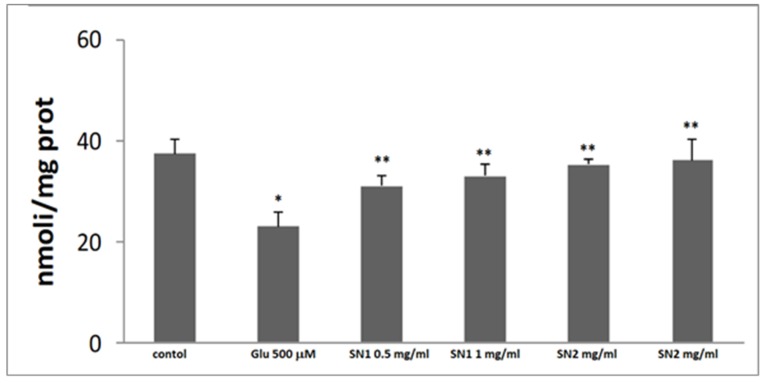
GSH levels in primary rat astroglial cell cultures at 14 DIV: exposed to glutamate 500 µM for 24 h. Bar 1: control. Bar 2: cell culture exposed 500 µM. Bar 3: cell culture exposed 500 µM plus SN1 0.5 mg/mL. Bar 4: cell culture exposed 500 µM plus SN1 1 mg/mL. Bar 5: cell culture exposed 500 µM plus SN2 0.5 mg/mL. Bar 6: cell culture exposed 500 µM plus SN2 1 mg/mL. Four replicates were carried out for each sample. (* *p* < 0.05 and ** *p* < 0.001). Axis: x label: concentration, y label: nmoli of GSH per mg of protein.

**Figure 4:**
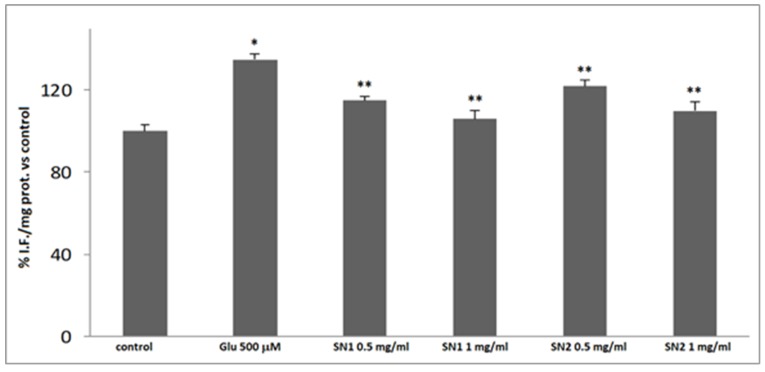
ROS levels in primary rat astroglial cell cultures at 14 DIV: exposed to glutamate 500 µM for 24 h. Bar 1: control. Bar 2: cell culture exposed 500 µM. Bar 3: cell culture exposed 500 µM plus SN1 0.5 mg/mL. Bar 4: cell culture exposed 500 µM plus SN1 1 mg/mL. Bar 5: cell culture exposed 500 µM plus SN2 0.5 mg/mL. Bar 6: cell culture exposed 500 µM plus SN2 1 mg/mL. Four replicates were carried out for each sample. (* *p* < 0.05 and ** *p* < 0.001). Axis: x label: concentrations, y label: percentage of fluorescence intensity per mg protein *versus* control.

The glutamate-evoked oxidative stress was evaluated by measuring the depletion of intracellular GSH levels (Figure 3) and ROS production (Figure 4). Glutamate (500 µM for 24 h) produced a significant decrease in the intracellular GSH levels and a significant increase of ROS levels, when compared to the untreated control ones. The pre-incubation of the cultures with SN1 and SN2 extracts (0.5 and 1 mg/mL) was able to restore, in a dose-dependent manner, GSH and ROS levels. In particular, 1 mg/mL of the extracts showed values similar to untreated control values.

## 4. Discussion

In this study, we assessed the antioxidant effect of SN1 and SN2 extracts of *Solanum nigrum* L. leaves, both in an in vitro cellular free system and in vitro cellular models.

Oxidative stress is the causative agent in a number of human diseases, such as atherosclerosis, ischemic reperfusion injury, inflammation, carcinogenesis, aging, and neurodegenerative diseases. Although there are many determinants in the development of these diseases, considerable experimental evidence links the production of ROS to biological damage that can potentially provide a mechanistic basis for their initiation and/or progression [36,37,38,39]. Moreover, because the ROS production is the fatal consequence of aerobic life, it is also an important component of the signaling network of plants [39], where polyphenols are the secondary metabolites produced as a defense mechanism against stress factors.

In this study, we exploited the sources, composition, and mechanisms of action of two polar *Solanum nigrum* L. leaf extracts natural products, and food, which represent a new frontier for therapy and a valuable tool to reduce the costs of health care systems.

In recent years, there has been great interest in the health effects of various natural products and in the in vivo protective function of natural antioxidants contained in dietary food against oxidative damage caused by ROS [40,41,42,43,44,45].

The free radical-scavenging activity is measured by the ability to bleach the stable DPPH radical. This assay provided information on the reactivity of test compounds with a stable free radical. SN1 and SN2 extracts were able to quench the DPPH-radical in a dose-dependent manner and showed comparable capacity. In fact, the two polar extracts content of different level phenolic components, but phenolic and flavones are not significantly different between SN1 and SN2. Only gentisic acid is more abundant in SN2. In addition, their effect appeared similar to Trolox. Then, this set of experiments demonstrate that SN1 and SN 2 possess comparable antioxidant properties.

Furthermore, we assessed the effect of the extracts in an in vitro cellular experimental model of excitotoxicity. In our previous research studies, using an experimental model of excitotoxicity, we demonstrated that glutamate exposure in primary cultures of astrocytes might be part of the biochemical response to oxidative stress induced by a prolonged exposure of astrocyte cultures to the neurotransmitter [46].

The antioxidant effect of the extracts SN1 and SN2 was also assessed in the cellular system using primary rat astroglial cell cultures exposed to the astroglial cell cultures in the presence of 500 μM glutamate for 24 hours. We used glutamate as a stressor because its high levels induce alterations in glutamate transport, mitochondria impairment, decrease ATP levels, GSH depletion, ROS production, macromolecular synthesis [35], and subsequent neuronal cell death [47,48].

Figure 2 shows the quenching of DPPH of SN1 and SN2 extracts at different concentrations, compared to Trolox, which shows a stronger activity at a lower concentration (0.025–0.5 and 0.1 mg/mL). In fact, we found that the extracts SN1 and SN2 were able to counteract the effect of glutamate, restoring, in a dose-dependent manner, the GSH and ROS levels similar to the control values. 

The statistical analysis method in this study indicated high significance (* *p* < 0.05 and ** *p* < 0.001) when compared with the control group, as reported in Figure 3 and Figure 4, where SN1 and SN2 extracts are compared with the cells exposed to glutamate 500 μM only.

The protective effect against glutamate toxicity of the extracts SN1 and SN2 appeared stronger than that of the synthetic antioxidant compounds used in our previous research studies [46].

Thus, these findings show that the extracts SN1 and SN2 possess antioxidant properties. Furthermore, it is possible to assume that the extracts of SN1 and SN2 of *Solanum nigrum* are able to counteract glutamate uptake-induced impairment of cystine/glutamate antiporter, which leads to depletion of the GSH content and biochemical alterations. This results in the delayed toxic effect for primary astroglial cell cultures [35]. Moreover, in a previous study, we reported that a pre-incubation with GYKI 52466, the selective inhibitor of AMPA/KA receptors, diminished glutamate effects, which indicates the involvement of receptor-linked events in GSH decrease and ROS increase levels [49].

Spectrophotometric and chromatographic analytical methods applied for estimation of total phenolic content and for determination of phenolic acids and flavones compounds in the examined extracts showed that these constituents are present in a valuable amount. Our results strongly suggest that phenolic compounds are important components of SN, and some of their pharmacological effects could be attributed to the presence of these compounds.

## 5. Conclusions

This study has provided some scientific rationale for these vegetables in the management of diseases, as obtained in folklore. 

SN leave extracts were able to reduce oxidative stress, and, in particular, they showed the potential in quenching the radical in vitro free cellular system, and restoring the oxidative status among in vitro primary rat astroglial cell cultures exposed to glutamate, which possesses notable antioxidant properties and neuroprotective effects. Furthermore, preventing the increase in glutamate uptake and inhibiting glutamate excitotoxicity, SN1 and SN2 leave polar extracts may represent a new natural therapeutic strategy in the neuro-pathological conditions associated with excitotoxicity.

Therefore, these vegetables may serve as a potential source of natural phenolic antioxidants for functional foods and nutraceuticals for the prevention and management of neurodegenerative diseases.

## Figures and Tables

**Table 1:** Extraction yields and concentration of total phenolic content of SN1 and SN2 extracts (*n* = 3).

Extract	Solvent	Physical	Yield %	Total Phenolic Content (mg/g of Extract)
SN1	MeOH-H_2_O 80–20 *v*/*v*	Brown solid	26.1 ± 3.9%	92.2 ± 4.8
SN2	H_2_O	Brown solid	19.5 ± 5.0%	40.0 ± 6.9

**Table 2:** Contents of phenolic acid and flavones (mg/g of dry weight) in SN1 and SN2 leave extracts (*n* = 3).

Extract	Gallic Acid	Protocatechuic Acid	Chlorogenic Acid	Gentisic Acid	Caffeic Acid	Luteolin	Apigenin
SN1	0.09 ± 0.02	0.24 ± 0.91	2.77 ± 0.45	1.50 ± 0.66	0.64 ± 0.87	0.98 ± 0.33	0.16 ± 0.74
SN2	0.04 ± 0.05	0.19 ± 0.11	2.01 ± 0.98	1.81 ± 0.75	0.42 ± 0.54	0.8 ± 0.62	0.12 ± 0.02

## Abbreviations

ATP	Adenosine Triphosphate
DCF	2′,7′-dichlorofluorescein
DCFH-DA	2′,7′-dichlorofluorescein diacetate
DIV	days in vitro
DMEM	Dulbecco’s Modified Eagle’s medium
DPPH	1,1-Diphenyl-2-Picrylhydrazyl radical
EDTA	ethylenediaminetetracetic acid
FBS	Heat Inactivated Fetal Bovine Serum
FITC	fluorescein isothiocyanate
GFAP	Glial Fibrillary Acidic Protein
GSH	reduced glutathione
HPLC	High performance liquid chromatography
MTT	3(4,5-dimethyl-thiazol-2-yl)2,5-diphenyl-tetrazolium bromide
NADH	β-Nicotinamide adenine dinucleotide
O_2_•^−^	superoxide anion
•OH	hydroxyl radical
ROO•	peroxyl radical
ROS	reactive oxygen species
SOD	Superoxide dismutase
SN	Solanum Nigrum L
XO	Xanthine oxidase

## References

[1] Särkinen T., Poczai P., Barboza G.E., van der Weerden G.M., Baden M., Knapp S. (2018). A revision of the Old World Black Nightshades (Morelloid clade of *Solanum* L., Solanaceae). PhytoKeys.

[2] Jagatheeswari D., Bharathi T., Sheik Jahabar Ali H. (2013). Black Night Shade (*Solanum nigrum* L.)-An Updated Overview. Int. J. Pharm. Biol. Arch..

[3] Akubugwo I.E., Obasi A.N., Ginika S.C. (2007). Nutritional Potential of the Leaves and Seeds of Black Nightshade-Solanum nigrum L. Var virginicum from Afikpo-Nigeria. Pak. J. Nutr..

[4] Leporatti M.L., Ghedira K. (2009). Comparative analysis of medicinal plants used in traditional medicine in Italy and Tunisia. J. Ethnobiol. Ethnomed..

[5] Jain R., Sharma A., Gupta S., Sarethy I.P., Gabrani R. (2011). *Solanum nigrum*: Current perspectives on therapeutic properties. Altern. Med. Rev..

[6] Wang Z., Li J., Ji Y., An P., Zhang S., Li Z. (2013). Traditional herbal medicine: A review of potential of inhibitory hepatocellular carcinoma in basic research and clinical trial. Evid. Based Complement. Altern. Med..

[7] Patel A., Biswas S., Shoja M.H., Ramalingayya G.V., Nandakumar K. (2014). Protective effects of aqueous extract of Solanum nigrum Linn. leaves in rat models of oral mucositis. Sci. World J..

[8] Wang C.K., Lin Y.F., Tai C.J. (2015). Integrated treatment of aqueous extract of *Solanum nigrum*-potentiated cisplatin- and doxorubicin-induced cytotoxicity in human hepatocellular carcinoma cells. Evid. Based Complement. Alternat. Med..

[9] Wang C.W., Chen C.L., Wang C.K. (2015). Cisplatin-, doxorubicin-, and docetaxel-induced cell death promoted by the aqueous extract of *Solanum nigrum* in human ovarian carcinoma cells. Integr. Cancer Ther..

[10] Tai C.J., Wang C.K., Chang Y.J., Lin C.S., Tai C.J. (2012). Aqueous extract of *Solanum nigrum* leaf activates autophagic cell death and enhances docetaxel-induced cytotoxicity in human endometrial carcinoma cells. Evid. Based Complement. Alternat. Med..

[11] Tai C.J., Wang C.K., Tai C.J. (2013). Aqueous extract of *Solanum nigrum* leaves induces autophage and enhances cytotoxicity of cisplatin, doxoorubicin, docetaxel, and 5-flurouracil in human colorectal carcinoma cells. Evid. Based Complement. Alternat. Med..

[12] Ikeda T., Tsumagari H., Nohara T. (2000). Steroidal oligoglycosides from Solanum nigrum. Chem. Pharm. Bull..

[13] Gu X.Y., Shen X.F., Wang L., Wu Z.W., Li F., Chen B., Zhang G.L., Wang M.K. (2018). Bioactive steroidal alkaloids from the fruits of Solanum nigrum. Phytochemistry.

[14] Xiang L., Wang Y., Yi X., He X. (2018). Anti-inflammatory steroidal glycosides from the berries of *Solanum nigrum L*. (European black nightshade). Phytochemistry.

[15] Esmaeili A., Tahazadeh A.R., Ebrahimzadeh M.A. (2017). Investigation of composition extracts, biological activities and optimization of *Solanum nigrum* L. extraction growing in Iran. Pak. J. Pharm. Sci..

[16] Wang H.C., Chung P.J., Wu C.H., Lan K.P., Yang M.Y., Wang C.J. (2011). *Solanum nigrum* L. polyphenolic extract inhibits hepatocarcinoma cell growth by inducing G2/M phase arrest and apoptosis. J. Sci. Food Agric..

[17] Huang H.C., Syu K.Y., Lin J.K. (2010). Chemical Composition of *Solanum nigrum* Linn Extract and Induction of Autophagy by Leaf Water Extract and Its Major Flavonoids in AU565 Breast Cancer Cells. J. Agric. Food Chem..

[18] Zaidi S.K., Hoda M.N., Tabrez S., Ansari S.A., Jafri M.A., Khan M.S., Hasan S., Alqahtani M.H., Abuzenadah A.M., Banu N. (2014). Protective Effect of *Solanum nigrum* Leaves Extract on Immobilization Stress Induced Changes in Rat’s Brain. Evid. Based Complement. Altern. Med..

[19] Upadhyay P., Ara S., Prakash P. (2015). Antibacterial and Antioxidant Activity of Solanum nigrum Stem and Leaves. Chem. Sci..

[20] Sun A.Y., Wang Q., Simonyi A., Sun G.Y., Benzie I.F.F., Sissi Wachtel-Galor S. (2011). Botanical Phenolics and Neurodegeneration. Herbal Medicine Biomolecular and Clinical Aspects.

[21] Ogunsuyi O.B., Ademiluyi A.O., Oboh G., Oyeleye S.I., Dada A.F. (2018). Green leafy vegetables from two Solanum spp. (*Solanum nigrum* L and *Solanum macrocarpon* L.) ameliorate scopolamine-induced cognitive and neurochemical impairments in rats. Food Sci. Nutr..

[22] Lobo V., Patil A., Phatak A., Chndra N. (2010). Free radicals, antioxidants and functional foods: Impact on human health. Pharmacogn. Rev..

[23] Zitka O., Skalickova S., Gumulec J., Masarik M., Adam V., Hubalek J., Trnkova L., Kruseova J., Eckschlager T., Kizek R. (2012). Redox status expressed as GSH:GSSG ratio as a marker for oxidative stress in paediatric tumour patients. Oncol Lett..

[24] Liu W., Duan X., Fang X., Shang W., Tong C. (2018). Mitochondrial protein import regulates cytosolic protein homeostasis and neuronal integrity. Autophagy.

[25] Walls A.B., Waagepetersen H.S., Bak L.K., Schousboe A., Sonnewald U. (2015). The glutamine glutamate/GABA cycle: Function, regional differences in glutamate and GABA production and effects of interference with GABA metabolism. Neurochem. Res..

[26] Devinsky O., Vezzani A., Najjar S., De Lanerolle N.C., Rogawski M.A. (2013). Glia and epilepsy: Excitability and inflammation. Trends Neurosci..

[27] Schousboe A., Bak L.K., Waagepetersen H.S. (2013). Astrocytic control of biosynthesis and turnover of the neurotransmitters glutamate and GABA. Front. Endocrinol..

[28] Agbor G.A., Vinson J.A., Donnelly P.E. (2014). Folin-Ciocalteau Reagent for Polyphenolic Assay. Int. J. Food Sci. Nutr. Diet..

[29] Sak K. (2014). Dependence of DPPH radical scavenging activity of dietary flavonoid quercetin on reaction environment. Mini-Rev. Med. Chem..

[30] Acquaviva R., Russo A., Galvano F., Galvano G., Barcellona M.L., Li Volti G., Vanella A. (2003). Cyanidin and cyanidin 3-O-beta-D -glucoside as DNA cleavage protectors and antioxidants. Cell Biol. Toxicol..

[31] Campisi A., Caccamo D., Raciti G., Cannavò G., Macaione V., Currò M., Macaione S., Vanella A., Ientile R. (2003). Glutamate-induced increases in transglutaminase activity in primary cultures of astroglial cells. Brain Res..

[32] Li Volti G., Ientile R., Abraham N.G., Vanella A., Cannavò G., Mazza F., Currò M., Raciti G., Avola R., Campisi A. (2004). Immunocytochemical localization and expression of heme oxygenase-1 in primary astroglial cell cultures during differentiation: Effect of glutamate. Biochem. Biophys. Res. Commun..

[33] Acquaviva R., Campisi A., Murabito P., Raciti G., Avola R., Mangiamel S., Musumeci I., Barcellona M.L., Vanella A., Li Volti G. (2004). Propofol attenuates peroxynitrite-mediated DNA damage and apoptosis in cultured astrocytes: An alternative protective mechanism. Anesthesiology.

[34] Murphy T.H., Baraban J.M. (1990). Glutamate toxicity in immature cortical neurons precedes development of glutamate receptor currents. Brain Res. Dev. Brain Res..

[35] Chen Y.H., Du G.H., Zhang J.T. (2000). Salvianolic acid B protects brain against injuries caused by ischemia-reperfusion in rats. Acta Pharmacol. Sin..

[36] Moloney J.N., Cotter T.G. (2018). ROS signalling in the biology of cancer. Semin. Cell Dev. Biol..

[37] Schieber M., Chandel N.S. (2014). ROS function in redox signaling and oxidative stress. Curr. Biol..

[38] Nistico S., Ventrice D., Dagostino C., Lauro F., Ilari S., Gliozzi M., Strongoli M.C., Vecchio I., Rizzo M., Mollace V., Muscoli C. (2013). Effect of MN (III) tetrakis (4-benzoic acid) porphyrin by photodynamically generated free radicals on SODs keratinocytes. J. Biol. Regul. Homeost. Agents.

[39] Del Río L.A. (2015). ROS and RNS in plant physiology: An overview. J. Exp. Bot..

[40] Carresi C., Musolino V., Gliozzi M., Maiuolo J., Mollace R., Nucera S., Maretta A., Sergi D., Muscoli S., Gratteri S. (2018). Anti-oxidant effect of bergamot polyphenolic fraction counteracts doxorubicin-induced cardiomyopathy: Role of autophagy and c-kitposCD45negCD31neg cardiac stem cell activation. J. Mol. Cell. Cardiol..

[41] Sharma A., Kaur M., Katnoria J.K., Nagpal A.K. (2018). Polyphenols in Food: Cancer Prevention and Apoptosis Induction. Curr. Med. Chem..

[42] Loffredo L., Perri L., Nocella C., Violi F. (2017). Antioxidant and antiplatelet activity by polyphenol-rich nutrients: Focus on extra virgin olive oil and cocoa. Br. J. Clin. Pharmacol..

[43] Rizzo M., Ventrice D., Giannetto F., Cirinnà S., Santagati N.A., Procopio A., Mollace V., Muscoli C. (2017). Antioxidant activity of oleuropein and semisynthetic acetyl-derivatives determined by measuring malondialdehyde in rat brain. J. Pharm. Pharmacol..

[44] Muscoli C., Lauro F., Dagostino C., Ilari S., Giancotti L.A., Gliozzi M., Costa N., Carresi C., Musolino V., Casale F. (2014). Olea Europea-derived phenolic products attenuate antinociceptive morphine tolerance: An innovative strategic approach to treat cancer pain. J. Biol. Regul. Homeost. Agents.

[45] Zhang Y., Li X., Wang Z. (2010). Antioxidant activities of leaf extract of Salvia miltiorrhiza Bunge and related phenolic constituents. Food Chem. Toxicol..

[46] Campisi A., Acquaviva R., Mastojeni S., Raciti G., Vanella A., De Pasquale R., Puglisi S., Iauk L. (2001). Effect of berberine and Berberis aetnensis C. Presl. alkaloid extract on glutamate-evoked tissue transglutaminase up-regulation in astroglial cell cultures. Phytother. Res..

[47] Pierozan P., Biasibetti H., Schmitz F., Ávila H., Parisi M.M., Barbe-Tuana F., Wyse A.T., Pessoa-Pureur R. (2016). Quinolinic acid neurotoxicity: Differential roles of astrocytes and microglia via FGF-2-mediated signaling in redox-linked cytoskeletal changes. Biochim. Biophys. Acta.

[48] Sandhu J.K., Pandey S., Ribecco-Lutkiewicz M., Monette R., Borowy-Borowski H., Walker P.R., Sikorska M. (2003). Molecular mechanisms of glutamate neurotoxicity in mixed cultures of NT2-derived neurons and astrocytes: Protective effects of coenzyme Q10. J. Neurosci. Res..

[49] Campisi A., Caccamo D., Li Volti G., Currò M., Parisi G., Avola R., Vanella A., Ientile R. (2004). Glutamate-evoked redox state alterations are involved in tissue transglutaminase upregulation in primary astrocyte cultures. FEBS Lett..

